# Structural Insight into Inhibition of CsrA-RNA Interaction Revealed by Docking, Molecular Dynamics and Free Energy Calculations

**DOI:** 10.1038/s41598-017-14916-6

**Published:** 2017-11-02

**Authors:** Xiaodong Ren, Rui Zeng, Micky Tortorella, Jinming Wang, Changwei Wang

**Affiliations:** 1 Department of Pharmacy, Guizhou Provincial People’s Hospital, Guiyang, 550002 P.R. China; 2 College of Pharmacy, Southwest University for Nationalities, Chengdu, 610041 P.R. China; 3 Guangzhou Institute of Biomedicine and Health (GIBH), Chinese Academy of Sciences (CAS), Guangzhou, Guangdong, 510530 P.R. China

## Abstract

The carbon storage regulator A (CsrA) and its homologs play an important role in coordinating the expression of bacterial virulence factors required for successful host infection. In addition, bacterial pathogens with deficiency of CsrA are typically attenuated for virulence. In 2016, the first series of small-molecule inhibitors of CsrA-RNA interaction were identified, which were found to achieve the CsrA-RNA inhibition by binding to the CsrA, without interfering with the RNA. However, the binding mechanism of these inhibitors of CsrA is not known. Herein, we applied molecular docking, molecular dynamics and binding free energy calculations to investigate the binding mode of inhibitors to CsrA. We found that the G_11_(RNA)-binding site is the most important binding site for CsrA inhibitors. An inhibitor with the proper size range can bind to that site and form a stable complex. We also found that inhibitors with larger size ranges bind to the entire CsrA-RNA interface, but have loose binding. However, this loose binding still resulted in inhibitory activity. The calculated binding free energy from MM/GBSA has a good correlation with the derived experimental binding energy, which might be used as a tool to further select CsrA inhibitors after a first-round of high-throughput virtual screening.

## Introduction

Bacterial adaptation to changing environments relies on the ability of the bacterial cell to coordinately regulate gene expression in response to chemical and physical signals by a variety of transcriptional and post-transcriptional regulation. The ribonucleic acid (RNA)-binding protein carbon storage regulator A (CsrA), which is also called regulator of secondary metabolism A or E (RsmA or RsmE) in some species are important and widespread post-transcriptional regulators^1–4^. CsrA recognises and binds to specific motifs in target mRNAs to regulate expression of genes for virulence factors^5,6^, quorum sensing^5,6^, motility^7,8^, carbon metabolism^9,10^, biofilm formation^11,12^, and peptide uptake^13^, etc.

Extensive studies demonstrated that CsrA and its homologs play an important role in coordinating the expression of bacterial virulence factors required for successful host infection^2,3^. Bacterial pathogens with deficiency in CsrA are typically attenuated for virulence, which is likely a result of gene expression misregulation and the resulting inability to make critical physiological transitions during an infection^2,3,14–17^. Hence, CsrA represents a promising anti-infective drug target.

**Figure 1:**
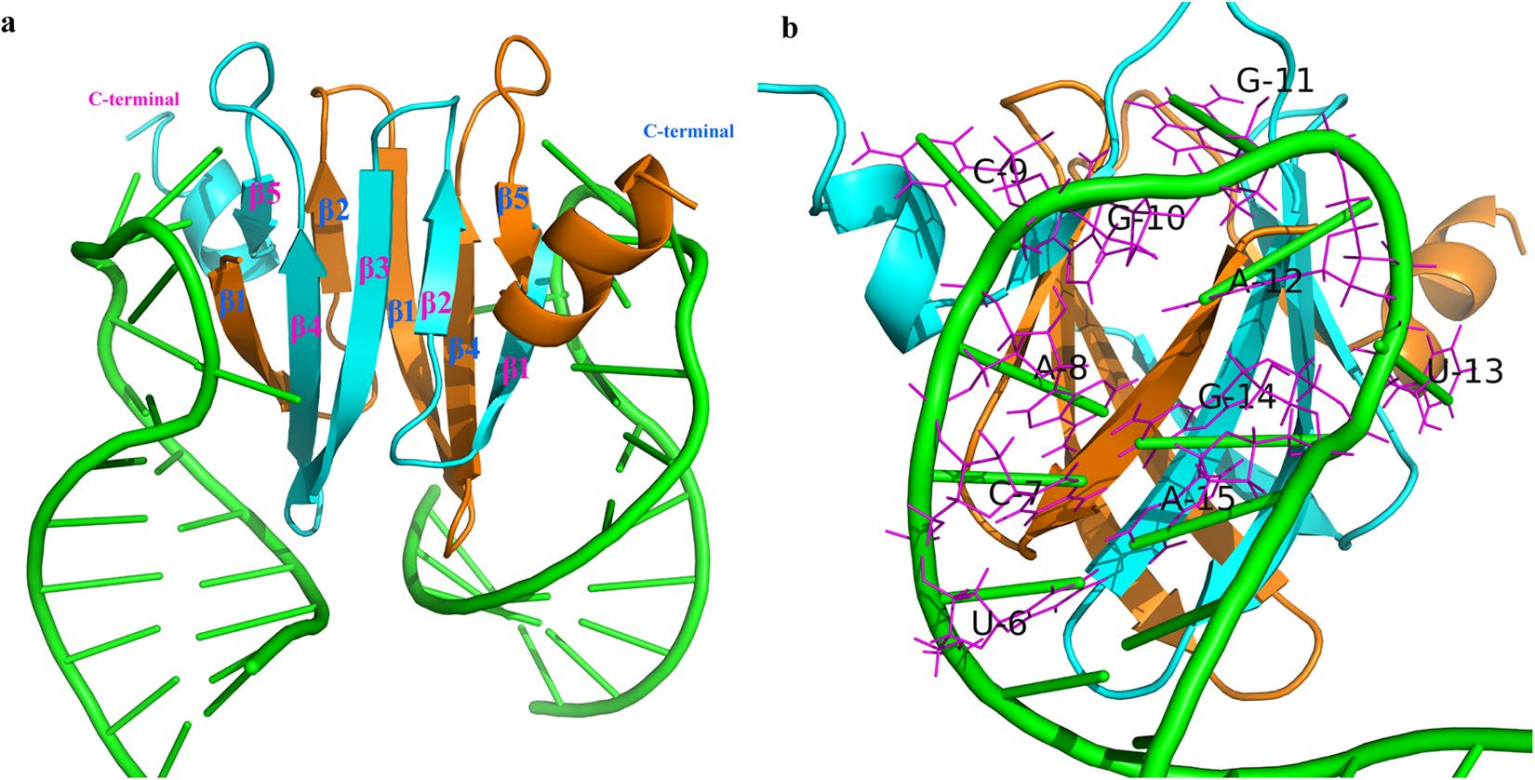
3D structure of the CsrA homologs RsmE binding with RNA (PDB ID: 2JPP). (**a**) The structure of the 2:2 complex of RsmE with 20-nucleotide *hcnA* RNA. Protein ribbons for each monomer are shown in orange and cyan. RNA cartoons are shown in green. (**b**) The structure of one RNA bound to the edge of the RsmE dimer with the second RNA molecule omitted in the background; the binding sequence motif UCACGGAUGA is shown by the magenta line.

The three-dimensional (3D) structures of CsrA and its homologs from different species have been solved previously, which demonstrated highly similar structures^18–23^. The 3D structure (Fig. 1) showed that two CsrA monomers, each composed of five β-strands and one α-helix, intertwine to form a symmetrical homodimer comprising a hydrophobic core and two identical RNA-binding surfaces^20^. The RNA-binding surfaces establish optimal contacts with a 5′-^A^/_U_CANGGANG^U^/_A_-3′ sequence motif present in the 5′ untranslated region (5′ UTR) of RNA^20,24^. When bound by CsrA, the ANGGA core folds into a loop stabilised by a 3-base pair (bp) stem of the flanking nucleotides. In this clamp-like structure, the Shine-Dalgarno sequence which is part of the ribosome-binding site and marks the starting point of translation, is sequestered and thus translation is repressed^25–27^. Small noncoding RNAs (sRNAs) that contain multiple CsrA binding sites antagonise CsrA in a competitive manner, which permits them to sequester multiple CsrA homodimers away from mRNA targets^28–30^.

**Figure 2:**
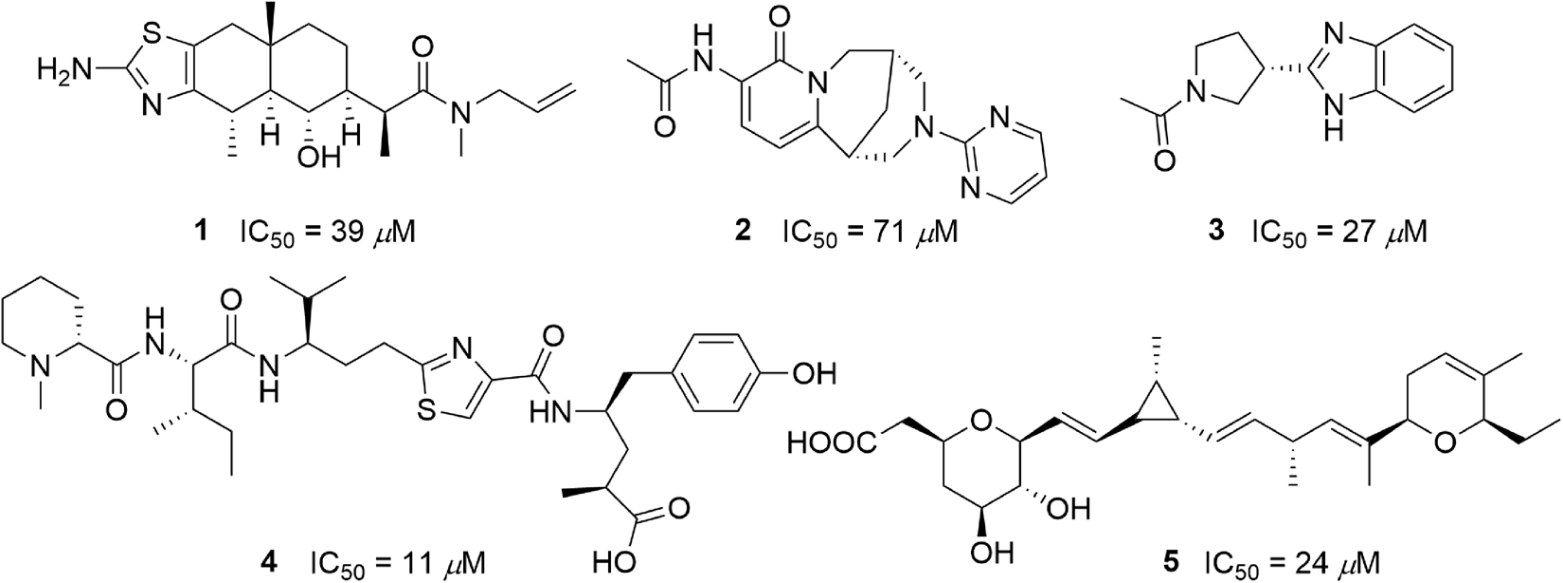
The structures and IC_50_ of the five CsrA inhibitors.

In 2016, Hartmann *et al*.^31^ described the discovery of the first CsrA-RNA interaction inhibitors by screening a library of small molecules. They identified seven structurally diverse hits capable of inhibiting the CsrA-RNA interaction in a dose-dependent manner with an IC_50_ range of 4 to 106 μM. Inhibition was achieved by dose-dependent binding of the inhibitor to CsrA and not by interfering with RNA. Five of the seven compounds are shown in Fig. 2, and structures of the other two compounds were not disclosed in Hartmann’s report for intellectual property reasons. As this is the first series of CsrA inhibitors to be identified, their binding mechanism is not known. Herein, we perform docking, molecular dynamics as well as free energy calculations to investigate the binding mechanism.

## Results

### Molecular docking

In the present study, we first used molecular docking to obtain the most possible binding pose for each compound. The five inhibitors were docked with CsrA at the RNA-binding interface. The predicted binding energy for each compound calculated by Autodock is summarised in Table 1. It was shown that despite compound **2** which has the lowest activity exhibited the highest binding energy, the ranking of the predicted binding energy for all the compounds was not in agreement with the experimental IC50 values reported.

**Table 1:** The binding energy of compounds binding to CsrA calculated by Autodock.

Compounds	IC_50_ (μM)	Binding energy(kcal/mol)
**1**	39	−7.86
**2**	71	−5.82
**3**	27	−5.90
**4**	11	−7.18
**5**	24	−7.78

**Figure 3:**
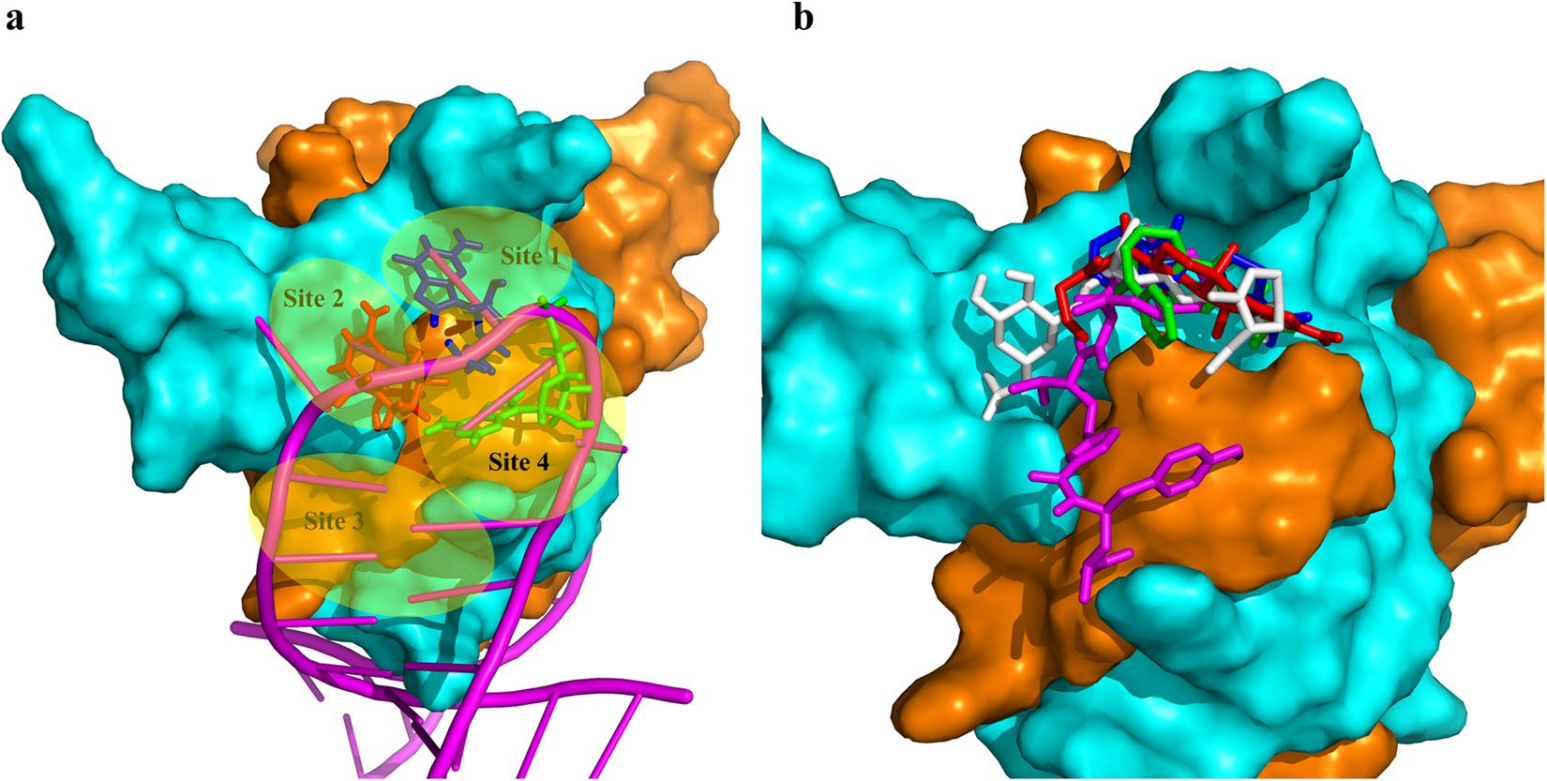
Potential binding sites on CsrA and docking binding pose of the inhibitors on the CsrA-RNA binding interface. (**a**) Potential binding sites on the CsrA-RNA binding interface. The interface was formed by the N-terminal of chain A (orange) and Chain B (cyan). RNA was shown as a magenta cartoon and the G_10_ (red), G_11_ (blue), A_12_ (green) of RNA core motif GGA are shown as sticks. (**b**) The binding pose of inhibitors of CsrA derived from Autodock. Compounds **1** (red), **2** (green), **3** (blue), **4** (magenta) and **5** (white) are shown as a stick.

To better study the binding poses, we analysed the RNA-binding surface of CsrA and designated four regions, namely sites 1 to 4 (Fig. 3a). As shown in Fig. 3a, the G_10_, G_11_, A_12_ of RNA core motif GGA in the complex occupied site 2, site 1 and site 4, respectively. The docking poses for each compound with the lowest binding energy which are the most likely binding poses, are shown in Fig. 3b. Compound **2** and **3** only occupy site 1, with their acetamide moiety oriented towards the N-terminal of chain A. Compound **5** occupies sites 1 and 2, with its carboxy group and tetrahydropyran moiety binding at site 2, while the dihydropyran end binding at site 1. Compound **1** mainly occupies site 1, but extends its allyl group into site 2. Due to its large size, compound **4**, which is the most active compound occupies sites 1 ~ 4.

### Stability of protein during MD simulation

**Figure 4:**
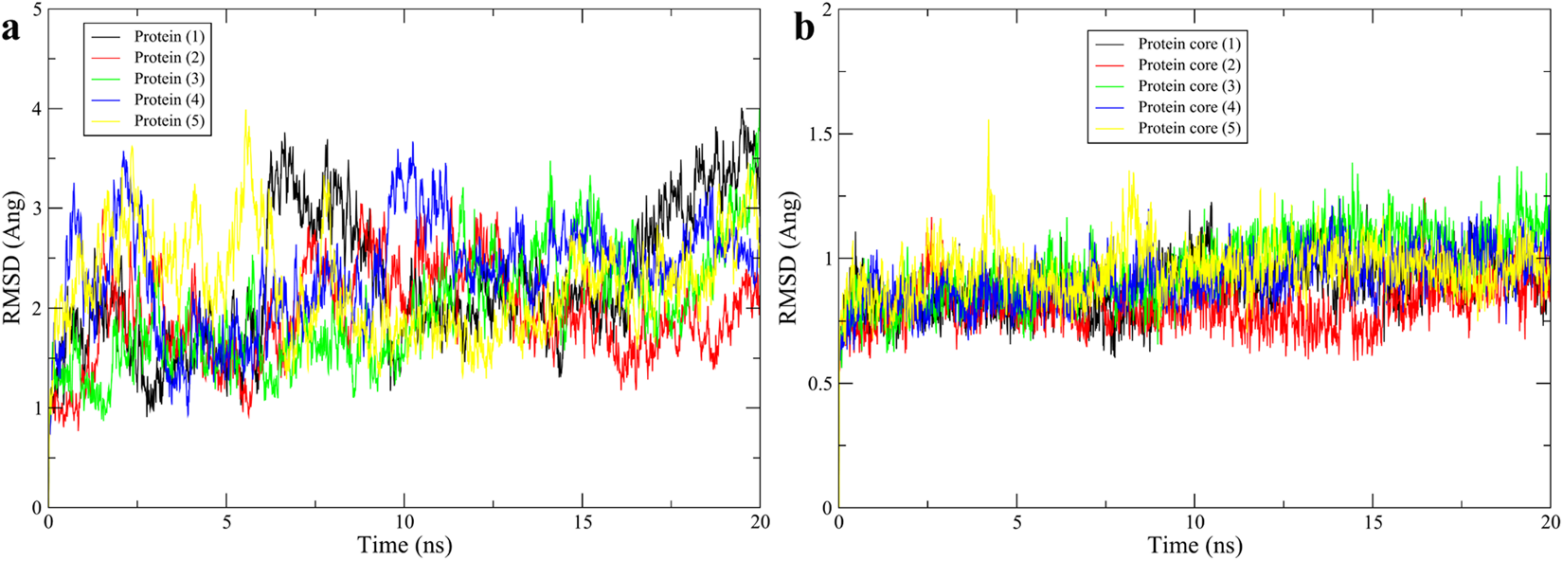
Backbone RMSD of the entire protein (**a**) and protein core structure (**b**). The number in the parentheses is compound number.

To investigate the thermodynamics of the complexes of CsrA and ligands, 20 ns MD simulation were performed for each complex. To monitor the structural stability of the receptor, the root mean square deviation (RMSD) values of the backbone atoms of the entire protein were calculated. As we envisioned that the C-terminal helix of both chains might have continuous fluctuation during the simulation, the backbone RMSD of protein cores (1–45 aa of both chains) without the C-terminal helix was calculated. As shown in the Fig. 4, the RMSD for the entire protein backbone in all complexes fluctuate continuously during the simulation, but the protein core without helixes in all complexes reached equilibrium after 8 ns of the simulation phase. As the binding sites are mainly located on the protein core structure, the trajectories of the MD simulations for all of the complexes after equilibrium of the protein core RMSD should be reliable for further analyses. It is noteworthy that the stability of the protein core does not necessarily guarantee the stability of the complexes as will be discussed later.

### Binding free energy calculated by the MM/GBSA method

The Molecular Mechanics/Generalized Born Surface Area (MM/GBSA) method has been widely utilised to study the receptor-ligand interaction in many cases^32^. In our study, 500 snapshots were extracted at every 10 ps of intervals from last 5 ns MD trajectory. Five different GB models available in AMBER (igb = 1 (GB^HCT^), 2 (GB^OBC I^), 5 (GB^OBC II^), 7 and 8; see *Methods* section) were used in this study, and the corresponding binding free energies are summarised in Table 2.

**Table 2:** MM/GBSA derived binding free energies of CsrA-inhibitor complexes calculated from the MD simulations using different GB Models.

Compounds	Δ*G*_MM-GBSA_^a^ (igb = 1)	Δ*G*_MM-GBSA_^a^ (igb = 2)	Δ*G*_MM-GBSA_^a^ (igb = 5)	Δ*G*_MM-GBSA_^a^ (igb = 7)	Δ*G*_MM-GBSA_^a^ (igb = 8)	IC_50_ (uM)	ΔG_calc_^a,b^
**1**	−19.81 ± 0.24	−15.74 ± 0.20	−15.83 ± 0.20	−14.73 ± 0.18	−13.66 ± 0.17	39	−6.05
**2**	−13.39 ± 0.26	−9.87 ± 0.23	−9.96 ± 0.24	−7.03 ± 0.20	−9.52 ± 0.20	71	−5.70
**3**	−21.12 ± 0.12	−17.51 ± 0.12	−18.40 ± 0.13	−12.48 ± 0.12	−13.80 ± 0.11	27	−6.27
**4**	−30.39 ± 0.18	−25.20 ± 0.17	−25.95 ± 0.18	−21.27 ± 0.17	−21.23 ± 0.16	11	−6.80
**5**	−16.20 ± 0.12	−18.07 ± 0.16	−19. 00 ± 0.17	−21.27 ± 0.19	−20.94 ± 0.18	24	−6.34

^a^All values are given in kcal/mol, and ΔG_MM-GBSA_ values are given as average ± SEM (standard error of the mean). ^b^Binding free energies ΔG_calc_ were calculated from experimental IC_50_ using the equation: Δ*G*_*calc*_ *≈ RT*ln IC_50_, where R is ideal gas constant, T is the temperature in K (300 K is used in this paper)^53,54^.

**Figure 5:**
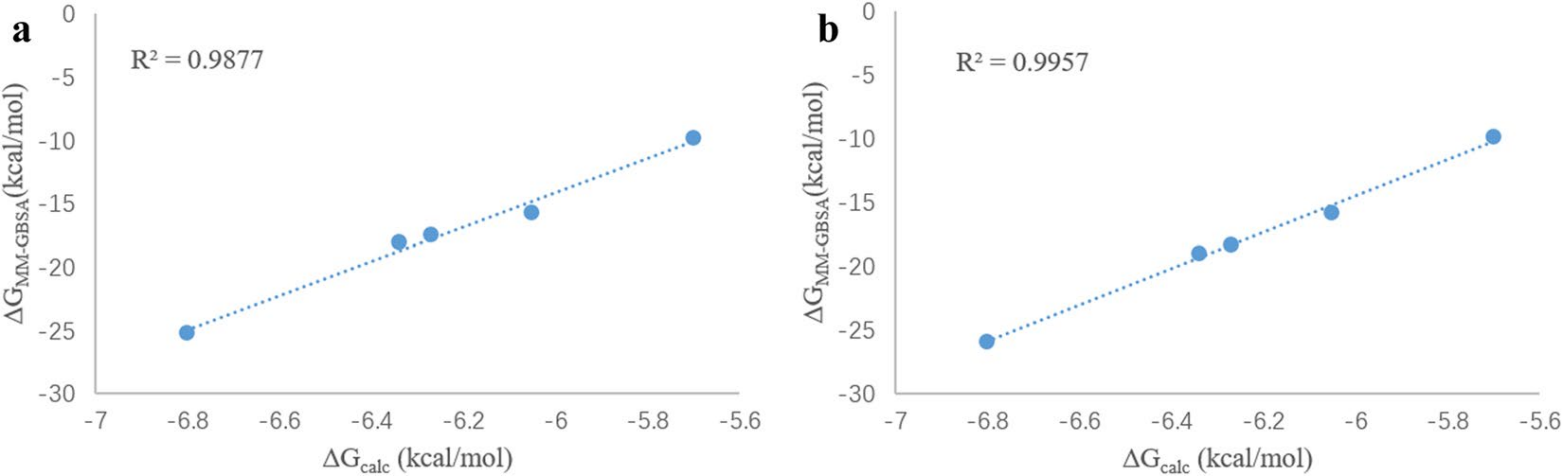
Correlation of the predicted binding energies by MM-GBSA (Δ*G*_MM-GBSA_) using GB^OBC I^ (**a**) and GB^OBC II^ models (**b**) with binding energy (Δ*G*_calc_) calculated from experimental IC_50_.

It was shown that there was a good correlation between predicted binding energies Δ*G*_MM-GBSA_ versus experimental IC_50_ derived binding energies Δ*G*_calc_. The correlation coefficient R^2^ for GB models GB^OBC I^ (igb = 2) and GB^OBC II^ (igb = 5) were 0.99 and 1.00 respectively (Fig. 5). The correlation coefficient when using igb = 1, 7 and 8 was 0.76, 0.74 and 0.79 respectively (The correlation graph for igb = 1, 7 and 8 can be found as Supplementary Figs S1–S3)

Table 3 showed different components of the Δ*G*_MM-GBSA_ using igb = 5 (The energies of different components of each complex using igb = 1, 2, 7 and 8 can be found as Supplementary Tables S1–S4). It was revealed that the polar component of solvation (Δ*G*_polar_) contributed unfavourably to binding of all five inhibitors, which are especially obvious in the **4** and **5** bound complexes. However, the unfavourable Δ*G*_polar_ is mostly, but not fully, compensated by the favourable electrostatic charge-charge interactions Δ*E*_ele_, especially in the **4** and **5** bound complexes.

**Table 3:** MM/GBSA binding free energies and different components of CsrA-inhibitor complexes calculated from the MD simulations using igb = 5.

Compounds	Δ*E*_vdW_^a^	Δ*E*_ele_^a^	Δ*G*_polar_^a^	Δ*G*_nonpolar_^a^	Δ*G*_MM-GBSA_^a^
**1**	−23.88 ± 0.23	−9.53 ± 0.23	20.50 ± 0.27	−2.92 ± 0.03	−15.83 ± 0.20
**2**	−15.95 ± 0.28	−21.14 ± 0.38	29.09 ± 0.43	−1.96 ± 0.03	−9.96 ± 0.24
**3**	−22.60 ± 0.10	−25.84 ± 0.36	32.71 ± 0.35	−2.67 ± 0.01	−18.40 ± 0.13
**4**	−27.92 ± 0.15	−78.78 ± 0.54	84.75 ± 0.54	−4.00 ± 0.02	−25.95 ± 0.18
**5**	−21.64 ± 0.12	−65.26 ± 0.70	71.34 ± 0.66	−3.44 ± 0.02	−19.00 ± 0.17

^a^All values are given in kcal/mol and as average ± SEM (standard error of the mean).

### Identification of the key residues

**Figure 6:**
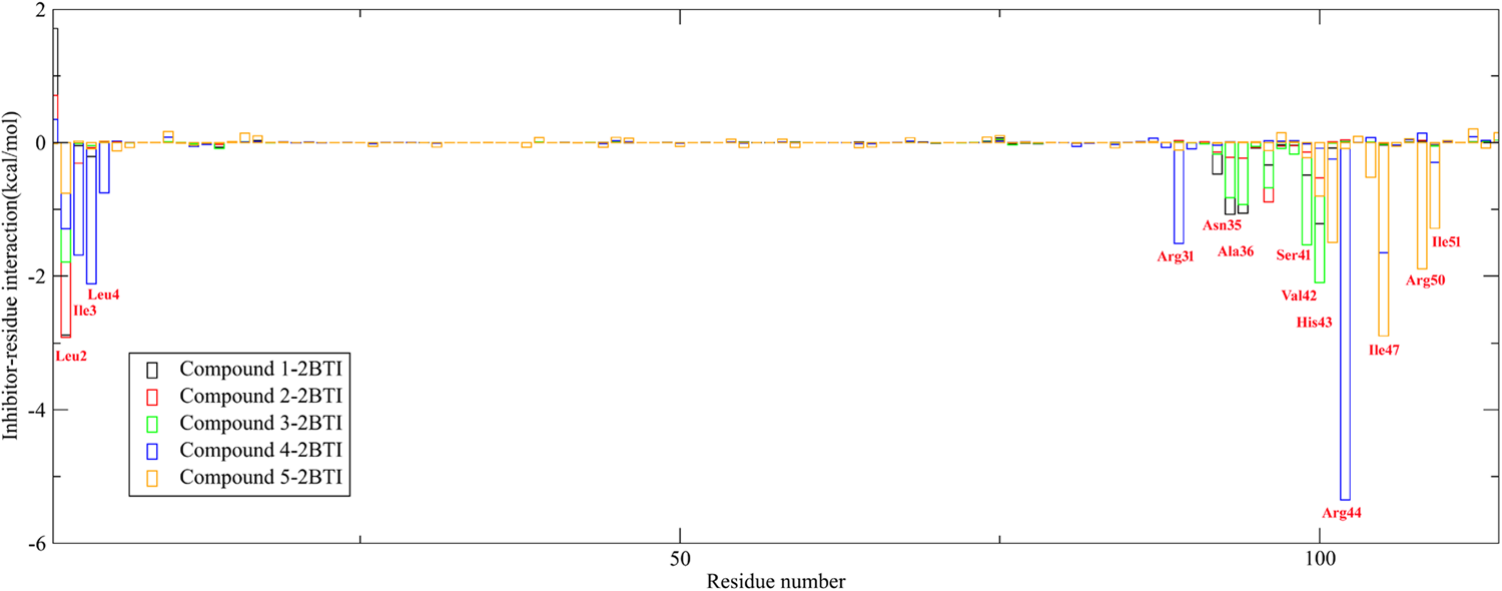
Contribution of each residue to the binding free energy. Residues 1–58 aa are residues comprising chain A, residues 59–144 aa represent chain B.

To obtain a more detailed thermodynamic description of the residue contributions to the binding free energy, we decomposed the binding energy Δ*G*_MM-GBSA_ on a per-residue level depicted in Fig. 6. As shown in Fig. 6, the contribution of an individual residue to binding varies from +1.7 to −5.4 kcal/mol. These groups of interactions consist of 13 residues in total with the binding energy of lower than −1 kcal/mol. The decomposition approach was helpful for locating residues that contribute to the receptor-ligand interaction.

For compound **1** and **3**, the binding energies were mainly due to residues Leu2(A), Asn35(B), Ala36(B), Ser41(B) and Val42(B). For compound **2**, the binding energies were guided by residues Leu2(A), Lys38(B). The residue contributions for compound **4** come from Leu2(A), Ile3(A), Leu4(A), Arg31(B) and Arg44(B), while the contributions for compound **5** arise from Val42(B), His43(B), Ile47(B), Arg50(B) and Ile51(B).

### Clustering analysis

**Figure 7:**
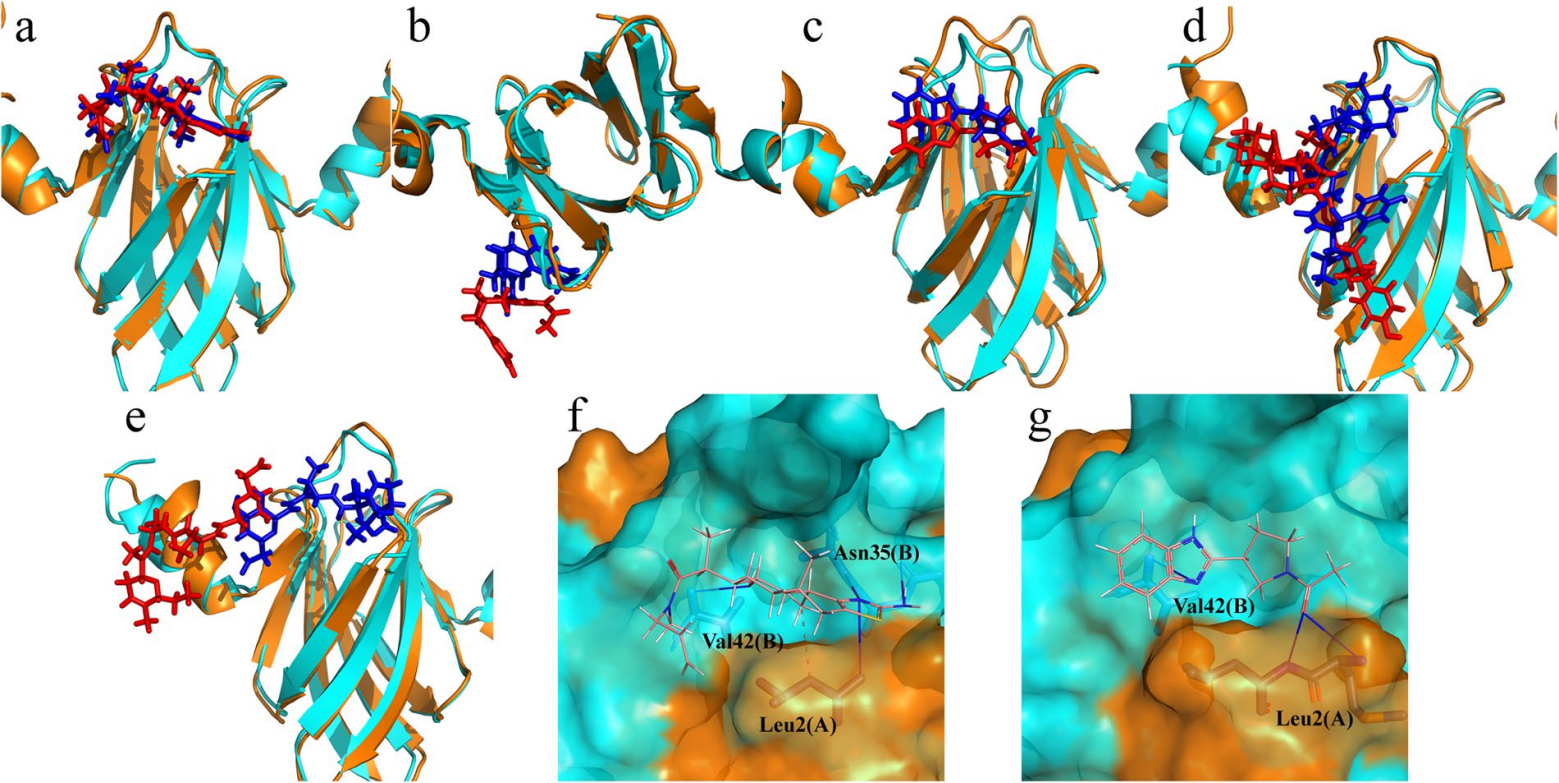
The conformation change of the complexes after MD simulation and receptor-ligand interaction. (**a**) ~ (**e**) are conformation change of the complexes of CsrA and compound **1** ~ **5** after MD simulation. For each comparison diagram, representative MD simulated CsrA (orange) and ligands (red) were aligned to the initial CsrA structure (cyan) and ligands (blue). (**f**) and (**g**) are binding site amino acid residue interactions between CsrA and bound **1** and **3**. Hydrogen bonds are depicted by a blue solid line, and hydrophobic interaction is depicted by a grey dotted line.

In order to explore the structural differences between the initial docking pose and the MD simulated pose, clustering analysis was applied to extract the representative conformation after 15 ns of MD simulation. Each trajectory of the last 5 ns was divided into five clusters using the average linkage algorithm. From the largest number of clusters, the conformation with the lowest RMSD to the cluster centre was selected. And the representative structures for each system compared with initial structures are shown in Fig. 7.

As shown in Fig. 7, the protein (especially the protein core structure without helixes) in all complexes are quite stable during simulation. Compound **1** and **3** still stayed in site 1, with only slight movement compared with the initial pose after 15 ns simulation. Compound **2** withdrew from site 1, which was probably the main reason that compound **2** exhibited an unfavourable binding free energy and poor *in vitro* activity. Compound **4** and **5** exhibited notable conformation changes, and both of them moved around the protein surface. Compound **4** mainly moved toward site 3 with its 1-methylpiperidine moiety withdrawn from site 1. Compound **5** also retreated from site 1 and moved toward the C-terminal of the chain B, and exhibited interactions with Arg50B and Ile51B at the C-terminus, as demonstrated in Fig. 6.

Among the five inhibitors, compound **1**, **2** and **3** have relative higher ligand efficiency (LE)^31^ of 0.24, 0.24 and 0.38 compared to compound **4** and **5**, with LE of 0.15 and 0.19. A higher LE is considered more favourable for affinity optimisation of a lead compound, as during the process of optimisation a lead molecule making its way to a clinical candidate, usually results in an further increase in molecular weight^33^. In addition, compound **4** and **5** have dramatic conformational or positional changes in comparison to its original pose. From the MD simulation results, compound **1** and **3** demonstrate the potential to be a lead compound for further optimisation. Although compound **2** has equal LE with **1**, its loose binding and poor activity make it hard to be optimised. We also analysed the residue interaction of CsrA with **1** and **3**. As shown in Fig. 7f ~ g, compound **1** forms one hydrogen bond with Leu2A and Val42B, two with Asn35B. In addition, compound **1** also formed a hydrophobic interaction with Leu2A. Compound **3** forms one hydrogen bond with Val42B and two hydrogen bonds with Leu2A. The binding interaction of compounds **1** and **3** with CsrA indicated that hydrogen bond formation is important to the binding, and compounds with hydrogen bond donor and acceptor potential with Leu2A, Asn35B and Val42B may result in improved activity.

## Discussion

Protein-protein or protein-RNA interactions are clearly challenging drug targets because the binding interface does not usually show small, deep cavities that are optimal for small-molecule-binding sites^34^. It is inspiring that the CsrA–RNA interaction could be blocked by small molecules like compound **3** with a low molecular weight (MW of 229 Da). As the first series of CsrA-RNA inhibitors to be identified, its binding mode to CsrA was not defined. Thus, the current study investigated the binding mechanism of these inhibitors to CsrA and may be helpful for better understanding the affinity differences between these molecules as well as help future drug design.

We firstly compared the sequence of CsrA from *Y. pseudotuberculosis* YPIII and the two chains in CsrA homolog RsmA from *Y. enterocolitica* (PDB ID: 2BTI), and the sequence alignment can be found as Supplementary Fig. S4. It was shown that CsrA bear only one residue difference (60^th^ aa) with the both chains of 2BTI. In protein 2BTI, 1–58 aa of chain A and 1–56 aa of chain B were solved, and the missing residues located in C-terminus, are far from the CsrA-RNA interface and not likely relevant. Thus, CsrA homolog RsmA proein was used in this study.

Molecular docking of the five inhibitors to the RNA-binding interface showed that site 1 (G_11_(RNA)-binding site) is the most viable site to accommodate small molecules, followed by site 2 (G_10_(RNA)-binding site), as evidenced by compounds **1** and **3** with their small molecular size binding to site 1, compound **4** with a larger size binds to both sites 1 and 2. Molecular docking is an important tool for obtaining possible binding poses for each compound; although, due to the structural diversity of the compounds and all the possible binding sites, the ranking of the predicted binding energy was not in agreement with the experimental IC50 values.

We also performed 20 ns MD simulation to study the thermodynamics of the complex, as well as calculated the binding free energy based on the MD trajectories. In the MM/GBSA free energy calculations, different GB models give slightly different polar solvation energies Δ*G*_*polar*_, which resulted in slightly different relative binding free energies Δ*G*_*MM-GBSA*._ The calculated binding free energies have good correlation with Δ*G*_*calc*_ which are derived from the experimental IC_50_ values. However, as the available data is limited, using the MM/GBSA method to more widely predict the binding energy and affinities still need more validation.

Clustering analysis provided important information and gave us a representative structure in a selected time period. The representative structures during the last 5 ns demonstrated different conformation changes. Compound **1** and **3** stayed in site 1 after at least 15 ns of simulation, showing that a small fragment binds to the site to form a stable complex and yield promising activity. Compound **2** retreated from the site 1 during simulation, indicating loose binding for compound **2** in site **1** resulting in decreased activity. Compound **4** and **5** have larger molecular sizes and can occupy more surfaces. In fact, compound **5** occupied both sites 1 and 2, while compound **4** occupied almost half of the interface, but readily moved around, indicating that there’s no deep pocket that can accommodate larger-size inhibitors. However, that does not necessarily mean the two inhibitors can more easily dissociate from the protein compared to compounds **1** and **3**, as demonstrated that **4** and **5** have the highest inhibitory activity among the five inhibitors.

From the point of view of medicinal chemistry, compound **3** with a simple structure, high ligand efficiency and obvious activity, may deserve further lead optimisation consideration.

In conclusion, we applied molecular docking, molecular dynamics and binding free energy calculations to investigate the binding mechanism of several inhibitors to CsrA and is the first report to study the binding of compounds to CsrA. We found that site 1 (G11(RNA)-binding site) is the most important binding site for small fragments. An inhibitor with proper size range and shape can bind to site 1 and form a stable complex. It was also found that inhibitors with large size range can bind to the entire interface, but are only loosely bound. However, this loose binding still resulted in promising inhibitory activity. The calculated binding free energy from MM/GBSA has a good correlation with the experimental inhibitory activity and might be used as a tool to further select CsrA inhibitors after a first-round high-throughput virtual screening. Our analysis in this report may facilitate further anti-infective drug design targeting CsrA.

## Methods

### Protein and ligand preparation

The CsrA protein used to screen the inhibitors in previous report^31^ was constructed based on CsrA gene from *Y. pseudotuberculosis* YPIII, and the 3D structure of CsrA from this species is not known. However, the structure of CsrA homolog RsmA (PDB ID: 2BTI), which only bear one resdue difference with CsrA, have been solved via X-ray crystallography^21^. Thus, the structure of RsmA was retrieved from the PDB bank(www.rcsb.org) and used in the following studies (We still called it CsrA in the whole study for clarity). The binding site was determined by aligning the structure of 2BTI to the RsmE/mRNA complex (PDB ID: 2JPP)^20^, as RsmE has 71% identity to CsrA (*Y. pseudotuberculosis* YPIII). All of the 3D structure of small molecules were built and energy minimised using Avogadro v1.2.0^35^. The steepest descent algorithm using the MMFF94 force field was used in the energy minimisation.

### Molecular Docking

AutoDock 4.2.3 program package was used for molecular docking and AutoDock Tools 1.5.6 (ADT) was used to prepare the PDBQT file format of ligands and protein^36^. The docking calculations were performed by locating a 50 × 90 × 80 points grid map and with a 0.375 Å grid point spacing which covers the entire interface of CsrA/RNA. 250 independent docking runs were performed for each docking simulation with 2,500,000 energy evaluations for each run. Other docking parameters were set to default. In docking calculations, the obtained poses were ranked using an energy-based scoring function. After all outputs were clustered based on the root mean squared deviation (RMSD) values, the top pose of docked ligands with the lowest energy in the biggest cluster were saved. For all docking analyses, only the best-scored pose was taken into account.

### Molecular Dynamics Simulation

Docked binding poses were used to run molecular dynamics simulations using the Amber16 software package^37^. Each compound was assigned AM1-BCC^38,39^ charges and gaff^40^ atom types using antechamber. Simulations were carried out using the GPU accelerated version of the PMEMD program with Amber ff99sb^41^ force field in periodic boundary conditions. Complexes were immersed in a truncated octahedron box of TIP3P^42^ water molecules with a margin distance of 12.0 Å. The solvated box was further neutralised with Na^+^ or Cl^−^ counter ions using the tleap program. Particle Mesh Ewald (PME)^43^ was employed to calculate the long-range electrostatic interactions. The cutoff distance for the long-range van der Waals (VDW) energy term was 12.0 Å. In order to remove any steric conflicts induced during system setup, structural optimisations were first performed on the relaxed water molecules and counter ions in two steps with the harmonic constraint potential of 2.0 kcal/mol·Å^2^ on all heavy atoms of both protein and ligands. Afterwards, the whole system was minimised without any restraint. The above steps were all executed by 2500 cycles of steepest descent minimization followed by 5000 cycles of conjugate gradient minimization. After system optimisation, running of MD simulations was started on the systems by gradually heating each system in the NVT ensemble from 0 to 300 K for 50 ps using a Langevin thermostat with a coupling coefficient of 1.0/ps and with a force constant of 2.0 kcal/mol·Å^2^ on the complex. And then 500 ps of density equilibration with a force constant of 2.0 kcal/mol·Å^2^ on the complex was performed. Subsequently, the systems were again equilibrated for 500 ps by releasing all the restraints. Finally, production runs for 20 ns MD simulations were performed under a constant temperature of 300 K in the NPT ensemble with periodic boundary conditions for each system. During the MD procedure, the SHAKE algorithm^44^ was applied for the constraint of all covalent bonds involving hydrogen atoms. The time step was set to 2 fs.

### Binding free energy calculations and per-residue free energy decomposition analysis

MM/GBSA free energy calculation has been successfully been used in many reported that can accurately predict the activity, which has the advantage of rapid speed, and the binding free energy can be decomposed into different components and on a per-residue level^32^.

The binding free energy was calculated using the MM/GBSA procedure implemented in Amber 16. The average 500 snapshots were extracted from the last 5 ns of MD trajectory at 10 ps intervals. Briefly, the MM/GBSA method can be summarised by the following equations.

1 $${\rm{\Delta }}{G}_{binding}={G}_{complex}-({G}_{receptor}+{G}_{ligand})$$

2 $${G}_{x}={E}_{MM}+{G}_{solv}-T{\rm{\Delta }}S$$

3 $${E}_{MM}={E}_{vdW}+{E}_{ele}$$

4 $${G}_{solv}={G}_{polar}+{G}_{nonpolar}$$

5 $${G}_{nonpolar}=\gamma SASA+\beta \,$$

6 $${\rm{\Delta }}{G}_{MM-GBSA}={\rm{\Delta }}{E}_{vdW}+{\rm{\Delta }}{E}_{ele}+{\rm{\Delta }}{G}_{polar}+{\rm{\Delta }}{G}_{nonpolar}$$

For each snapshot, binding free energy was calculated as the difference between the free energy of the complex (*G*_*complex*_) and the total of the free energies of the receptor (*G*_*receptor*_) and the ligand (*G*_*ligand*_), shown in equation (1). The free energy of each component *G*_*x*_ in equation (1) can be computed as the sum of the molecular mechanical(MM) gas-phase binding energy (*E*_*MM*_), the solvation free energy (*G*_*solv*_) and the configurational entropy (−*T*Δ*S*) contribution (equation (2)). *E*_*MM*_ is further divided into van der Waals (*E*_*vdW*_) and gas-phase electrostatic energies (*E*_*ele*_) (equation (3)), while the solvation free energy (*G*_*solv*_) is further divided into a polar (*G*_*polar*_) and a nonpolar (*G*_*nonpolar*_) component (equation (4)).

The polar solvation energy contribution was calculated by solving Generalized Born (GB) equation (MM-GBSA calculation) with the MM-GBSA module in AMBER. There are five different GB models, namely GB^HCT^(igb = 1)^45^, GB^OBC I^ (igb = 2)^46^, GB^OBC II^ (igb = 5)^46^, and two GBn models (igb = 7, 8)^47^ developed until now. In our study, all of the five available GB solvation models are used to see which one can best predict the activity of inhibitors. The nonpolar component (*G*_*nonpolar*_) was determined using equation (5), where SASA is the solvent-accessible surface area, with the γ and β using the default value. The value of the implicit solvent dielectric constant and the solute dielectric constant for GB calculations was set to 80 and 1, respectively. The solvent probe radius was set to 1.4 Å as default.

The absolute binding energy is often determined by considering the conformational entropy contribution (−*T*Δ*S*), where T is the absolute temperature and S the entropy of the molecule. The entropy of the molecule accounts for the loss of translational, rotational and conformational degrees of freedom of ligand upon binding. It has already been reported that inclusion of entropy in calculations did not always improve the accuracy^48^. It was also found that the inclusion of conformational entropy compromised the agreement between predicted absolute binding free energy and experimental binding free energy due to large fluctuations in the calculated entropy values^49^. In addition, the entropy calculation is computationally expensive. Thus, the binding free energy was calculated without considering the entropy contribution in this study to see whether a correlation could be achieved between relative binding free energies and biological data.

In summary, the relative binding energy (Δ*G*_*MM-GBSA*_) are evaluated by a sum of the changes of each component in equation (6).

To obtain a detailed view of the protein-ligand binding and identify the key residues responsible for the binding, free energy decomposition to each residue was performed using the MM/GBSA method with GB^OBC I^ model (igb = 2). All energy decomposition analyses were performed on the same snapshots which were used in the above calculations.

### Trajectory analysis

Clustering is a means of partitioning data so that data points inside a cluster are more similar to each other than they are to points outside a cluster. The cluster analysis of protein conformations was carried out using cpptraj module with average linkage as the clustering algorithm, and backbone atom RMSD as the distance metric. The average linkage algorithm is recommended in the previous report^50^. The interactions between ligand atoms and protein residues were determined using the fully automated protein–ligand interaction profiler (PLIP)^51^. Pymol 1.8^52^ was used for structural alignments and visualisations. For plotting graphs, MS Excel (2016), Xmgrace (Grace 5.1.25) were used.

## Electronic supplementary material

Supplementary Information
