# Supplementary material for: Structural Insight into Inhibition of CsrA-RNA Interaction Revealed by Docking, Molecular Dynamics and Free Energy Calculations
Source: Sci Rep. 2017 Nov 2;7:14934. doi: 10.1038/s41598-017-14916-6 (PMC5668361; doi:10.1038/s41598-017-14916-6)
Supplement: Supplementary file 1 — Supplementary Information [file 41598_2017_14916_MOESM1_ESM.pdf]

Supplementary Information

Structural Insight into Inhibition of CsrA-RNA Interaction Revealed by Docking, Molecular Dynamics and Free Energy Calculations

Xiaodong Ren[1], Rui Zeng[2], Micky Tortorella[3], Jinming Wang[3] & Changwei Wang[3,*]

[1]Department of Pharmacy, Guizhou Provincial People's Hospital, Guiyang 550002, P.R. China.

[2]College of Pharmacy, Southwest University for Nationalities, Chengdu 610041, P.R. China.

[3]Guangzhou Institute of Biomedicine and Health (GIBH), Chinese Academy of Sciences (CAS), Guangzhou, Guangdong 510530, P.R. China

Correspondence and requests for materials should be addressed to C.W. (email: wang_changwei@gibh.ac.cn)

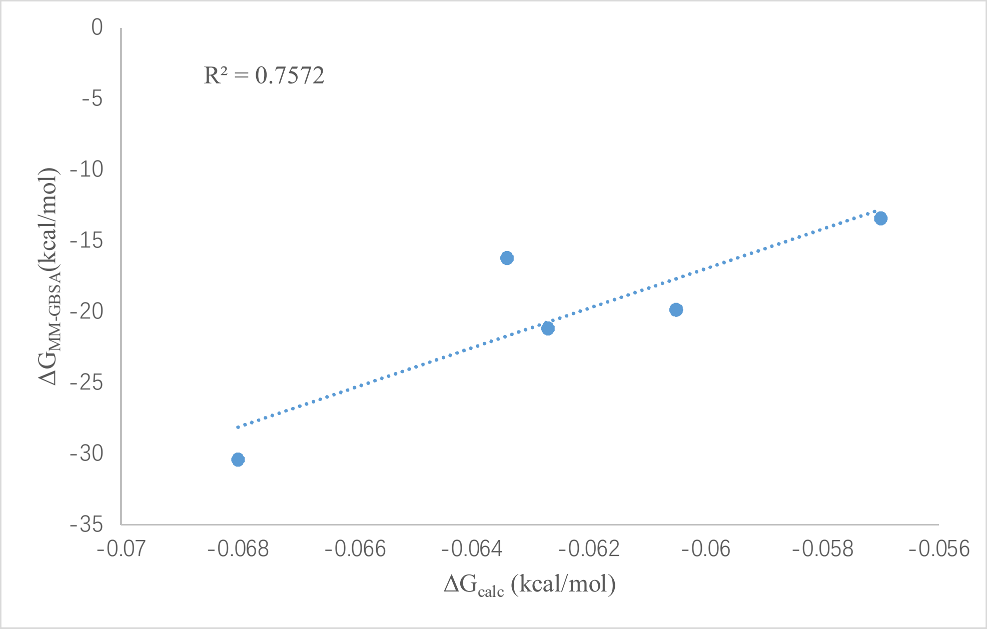

Figure S1. Correlation of the predicted binding energies by MM-GBSA ($\Delta G_{MM\text{-}GBSA}$) using igb = 1 with binding energy (ΔG_{calc}) calculated from experimental IC_{50}.

………

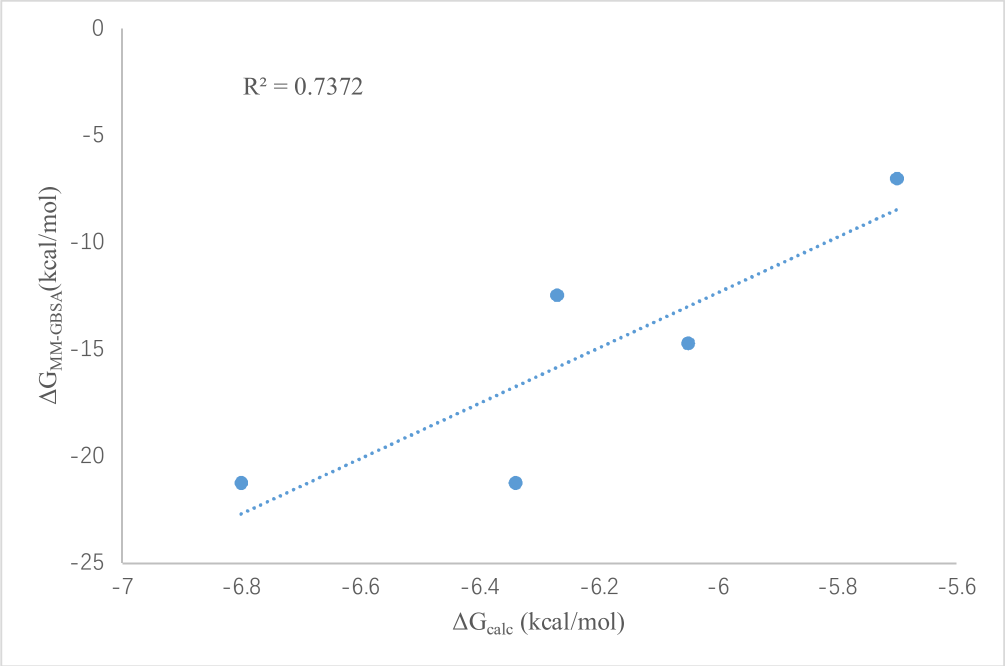

Figure S2. Correlation of the predicted binding energies by MM-GBSA ($\Delta G_{MM\text{-}GBSA}$) using igb = 7 with binding energy (ΔG_{calc}) calculated from experimental IC_{50}.

………

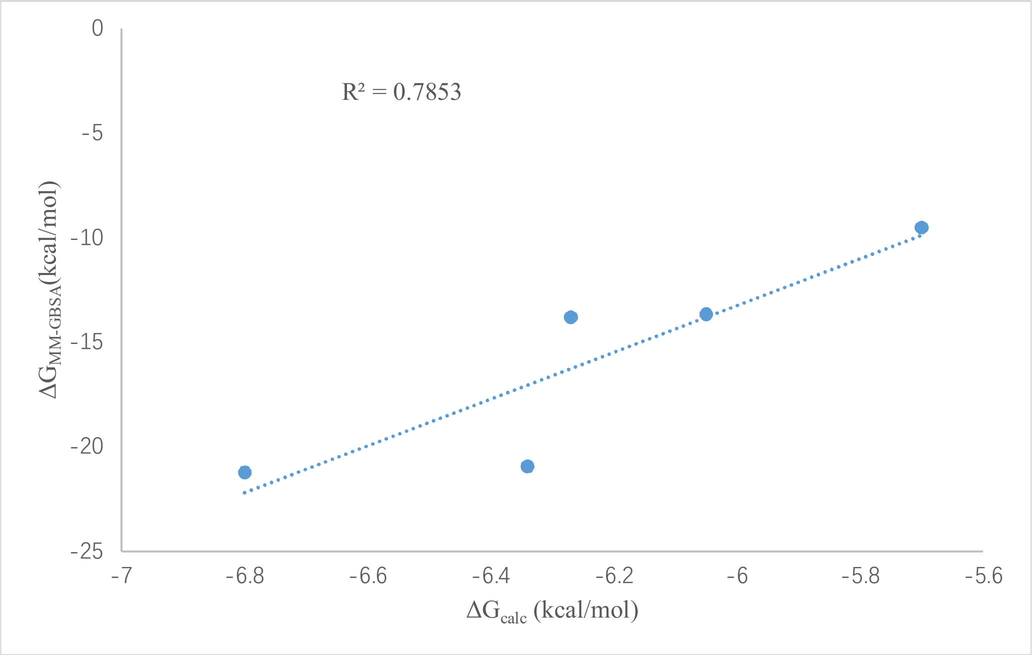

Figure S3. Correlation of the predicted binding energies by MM-GBSA ($\Delta G_{MM\text{-}GBSA}$) using igb = 8 with binding energy (ΔG_{calc}) calculated from experimental IC_{50}.

```
                                    10         20         30         40         50         60
                            ....|....| ....|....| ....|....| ....|....| ....|....| ....|....| .
CsrA(Y. pseudotuberculosis) MLILTRRVGE TLMIGDEVTV TVLGVKGNQV RIGVNAPKEV SVHREEIYQR IQAEKSQPTT Y
2BTI_A                      MLILTRRVGE TLMIGDEVTV TVLGVKGNQV RIGVNAPKEV SVHREEIYQR IQAEKSQPTS Y
2BTI_B                      MLILTRRVGE TLMIGDEVTV TVLGVKGNQV RIGVNAPKEV SVHREEIYQR IQAEKSQPTS Y
```

Figure S4. Sequence alignment between CsrA from *Y. pseudotuberculosis* YPIII and the two chains of 2BTI[a].

[a]Note: The amino acid sequence of CsrA (*Y. pseudotuberculosis*) was retrived from UniProt database (Accession No. B1JJ99) at http://www.uniprot.org. The sequence of 2BTI protein are retrived from Protein Data Bank (PDB). Sequence alignment was carried out using Pymod 2.0[1]. The aligned sequences are plotted using BioEdit 7.2.5[2].

Table S1. MM/GBSA binding free energies and different components of CsrA-inhibitor complexes calculated from the MD simulations using igb = 1. [a]All values are given in kcal/mol and as average ± SEM (standard error of the mean).

Compounds	ΔE_{vdW} [a]	ΔE_{ele} [a]	ΔG_{polar} [a]	$\Delta G_{nonpolar}$ [a]	$\Delta G_{MM\text{-}GBSA}$ [a]
1	-23.88 ± 0.23	-9.53 ± 0.23	16.52 ± 0.22	-2.92 ± 0.03	-19.81 ± 0.24
2	-15.95 ± 0.28	-21.14 ± 0.38	25.66 ± 0.40	-1.96 ± 0.03	-13.39 ± 0.26
3	-22.60 ± 0.10	-25.84 ± 0.36	29.99 ± 0.34	-2.67 ± 0.01	-21.12 ± 0.12
4	-27.92 ± 0.15	-78.78 ± 0.54	80.31 ± 0.53	-4.00 ± 0.02	-30.39 ± 0.18
5	-21.64 ± 0.12	-65.26 ± 0.70	74.14 ± 0.71	-3.44 ± 0.02	-16.20 ± 0.12

Table S2. MM/GBSA binding free energies and different components of CsrA-inhibitor complexes calculated from the MD simulations using igb = 2. [a]All values are given in kcal/mol and as average ± SEM (standard error of the mean).

Compounds	ΔE_{vdW} [a]	ΔE_{ele} [a]	ΔG_{polar} [a]	$\Delta G_{nonpolar}$ [a]	$\Delta G_{MM\text{-}GBSA}$ [a]
1	-23.88 ± 0.23	-9.53 ± 0.23	20.59 ± 0.27	-2.92 ± 0.03	-15.74 ± 0.20
2	-15.95 ± 0.28	-21.14 ± 0.38	29.18 ± 0.44	-1.96 ± 0.03	-9.87 ± 0.23
3	-22.60 ± 0.10	-25.84 ± 0.36	33.60 ± 0.35	-2.67 ± 0.01	-17.51 ± 0.12
4	-27.92 ± 0.15	-78.78 ± 0.54	85.50 ± 0.54	-4.00 ± 0.02	-25.20 ± 0.17
5	-21.64 ± 0.12	-65.26 ± 0.70	72.27 ± 0.67	-3.44 ± 0.02	-18.07 ± 0.16

………..

Table S3. MM/GBSA binding free energies and different components of CsrA-inhibitor complexes calculated from the MD simulations using igb = 7. [a]All values are given in kcal/mol and as average ± SEM (standard error of the mean).

Compounds	ΔE_{vdW} [a]	ΔE_{ele} [a]	ΔG_{polar} [a]	$\Delta G_{nonpolar}$ [a]	$\Delta G_{MM\text{-}GBSA}$ [a]
1	-23.88 ± 0.23	-9.53 ± 0.23	21.60 ± 0.30	-2.92 ± 0.03	-14.73 ± 0.18
2	-15.95 ± 0.28	-21.14 ± 0.38	32.02 ± 0.49	-1.96 ± 0.03	-7.03 ± 0.20
3	-22.60 ± 0.10	-25.84 ± 0.36	38.63 ± 0.39	-2.67 ± 0.01	-12.48 ± 0.12
4	-27.92 ± 0.15	-78.78 ± 0.54	89.43 ± 0.54	-4.00 ± 0.02	-21.27 ± 0.17
5	-21.64 ± 0.12	-65.26 ± 0.70	69.07 ± 0.65	-3.44 ± 0.02	-21.27 ± 0.19

………..

Table S4. MM/GBSA binding free energies and different components of CsrA-inhibitor complexes calculated from the MD simulations using igb = 8. [a]All values are given in kcal/mol and as average ± SEM (standard error of the mean).

Compounds	ΔE_{vdW} [a]	ΔE_{ele} [a]	ΔG_{polar} [a]	$\Delta G_{nonpolar}$ [a]	$\Delta G_{MM\text{-}GBSA}$ [a]
1	-23.88 ± 0.23	-9.53 ± 0.23	22.67 ± 0.30	-2.92 ± 0.03	-13.66 ± 0.17
2	-15.95 ± 0.28	-21.14 ± 0.38	29.53 ± 0.48	-1.96 ± 0.03	-9.52 ± 0.20
3	-22.60 ± 0.10	-25.84 ± 0.36	37.31 ± 0.37	-2.67 ± 0.01	-13.80 ± 0.11
4	-27.92 ± 0.15	-78.78 ± 0.54	89.47 ± 0.54	-4.00 ± 0.02	-21.23 ± 0.16
5	-21.64 ± 0.12	-65.26 ± 0.70	69.40 ± 0.65	-3.44 ± 0.02	-20.94 ± 0.18

References

1 Janson, G., Zhang, C., Prado, M. G. & Paiardini, A. PyMod 2.0: improvements in protein sequence-structure analysis and homology modeling within PyMOL. *Bioinformatics* **33**, 444-446,(2017).

2 Hall, T. A. BioEdit: a user-friendly biological sequence alignment editor and analysis program for Windows 95/98/NT. *Nucl. Acids. Symp. Ser.* **41**, 95-98,(1999).
